# Successful treatment of nail psoriasis with topical roflumilast: A case report

**DOI:** 10.1177/2050313X241289594

**Published:** 2024-10-22

**Authors:** Leah A Johnston, Susan M Poelman

**Affiliations:** 1 Cumming School of Medicine, University of Calgary, Calgary, AB, Canada; 2 Faculty of Medicine, University of Ottawa, Ottawa, ON, Canada; 3 Division of Dermatology, University of Calgary, Calgary, AB, Canada; 4 Beacon Dermatology, Calgary, AB, Canada

**Keywords:** Nail psoriasis, psoriatic nails, psoriasis, roflumilast, topical phosphodiesterase-4 inhibitor, inflammatory nail dystrophy

## Abstract

Nail psoriasis occurs in approximately half of all cases of plaque psoriasis and manifests with onychodystrophy, which includes morphological features of onycholysis, subungual hyperkeratosis, oil drop sign, pitting, splinter hemorrhages, leukonychia, and crumbling of the nails. Nail psoriasis can have a significant adverse impact on quality of life. However, nail psoriasis is often refractory to both local and systemic therapies, making it challenging to treat. Topical and oral phosphodiesterase-4 inhibitors have been successfully used to treat multiple different subtypes of psoriasis. Topical roflumilast, a phosphodiesterase-4 inhibitor cream, has recently received United States Food and Drug Administration and Health Canada approval for the treatment of plaque psoriasis. In this case report, a 25-year-old female with a 20-year history of nail psoriasis achieved complete resolution of her onychodystrophy after 5 months of daily application of topical roflumilast, without experiencing any side effects. This case report suggests that topical roflumilast may be a useful and well-tolerated therapy for psoriatic nails.

## Introduction

Nail psoriasis affects approximately 50% of all patients with plaque psoriasis, which has an overall prevalence of 2.5% in Canada.^1,2^ Potential triggers of psoriatic nails include local trauma and nail infections, and nail involvement occurs more frequently in individuals with psoriatic arthritis.^1^ Clinical manifestations of psoriatic nails include onycholysis, subungual hyperkeratosis, oil drop sign, pitting, splinter hemorrhages, leukonychia, and crumbling of the nails.^2^ The pathogenesis of nail psoriasis is thought to be similar to plaque psoriasis, which involves an interleukin (IL)-23/T helper 17-mediated increase in IL-2, IL-6, IL-8, IL-12, IL-17, IL-22, IL-23, interferon (IFN)-γ, tumor necrosis factor (TNF)-α, and nuclear factor-kappa B (NF-κB).^3–6^ However, IL-10, an anti-inflammatory cytokine, is downregulated in plaque psoriasis but has been found to be upregulated in psoriatic nails, which may be the result of anatomic differences between the skin and nails that lead to local variations in innate immune system activity.^6,7^ The anatomic structure and location of the nails also cause nail psoriasis to be notoriously challenging to treat.^8^ In this case report, we present a patient with nail psoriasis who was successfully treated with topical roflumilast, a phosphodiesterase-4 (PDE4) inhibitor.

## Case report

A 25-year-old female presented to the clinic with a 20-year history of onychodystrophy that affected the right third fingernail. Additionally, she described tenderness to palpation and sensations of pressure and tightness of the skin surrounding the nailbed. She had a history of similar findings on the fourth right finger, which subsequently resolved without intervention. The patient denied any history of trauma to the fingernail or previous infections but noted that she had sucked on her fingers during early childhood. The patient was otherwise well and did not have any history of other psoriatic lesions or arthralgias.

On examination, substantial onychodystrophy with onycholysis, a salmon-colored oil drop, and subungual hyperkeratosis of the right third fingernail was observed, along with scaling and erythema of the cuticle, proximal and lateral nail folds, and the distal pulp of the surrounding finger. A clinical diagnosis of nail psoriasis was made, and the patient was subsequently started on daily topical application of 0.3% roflumilast cream. After 5 months of using topical roflumilast, the patient had complete resolution of pain, erythema, and scaling of the skin surrounding the nail and complete regrowth of the affected nail without onychodystrophy. No side effects were reported.

## Discussion

This case demonstrates that topical roflumilast may be beneficial in managing nail psoriasis. Existing treatments for psoriatic nails include topical and locally injected corticosteroids, topical vitamin D analogs, topical calcineurin inhibitors, retinoids, phosphodiesterase inhibitors, oral corticosteroids, methotrexate, cyclosporine, phototherapy, and biologic therapies.^9^ Due to the chronic, relapsing, and often recalcitrant nature of nail psoriasis, a combination of local and systemic modalities is often needed to achieve remission.^9^ Nail psoriasis and its variants are notoriously refractory to topical therapies, as the thick and rigid keratin network of the nail plate limits the diffusion of topical drugs into the underlying nail bed.^8^ In this case, the patient achieved complete remission of fingernail psoriasis through monotherapy with topical roflumilast.

Topical 0.3% roflumilast cream has been recently approved by the United States Food and Drug Administration and Health Canada for the treatment of chronic plaque psoriasis.^10^ Phase 2 and 3 clinical trials have demonstrated high efficacy and tolerability of topical roflumilast throughout long-term periods of daily application for up to 64 weeks.^10^ Roflumilast inhibits the activity of PDE4, subsequently inducing an upregulation in cyclic adenosine monophosphate and causing downstream reductions in the activity of NF-κB and levels of IL-17, IL-23, IFN-γ, and TNF-α.^10^

The use of topical roflumilast to treat nail psoriasis has not been explored in randomized controlled trials but may be a useful consideration for future research and drug development. While topical roflumilast is currently marketed as a cream formulation (Arcutis Biotherapeutics, Inc.), roflumilast may be more effective for treating nail psoriasis in the form of a nail lacquer.^11,12^ A 2017 study evaluated the use of another PDE4 inhibitor, topical apremilast (1 mg/ml), compounded with a methacrylic acid and methyl methacrylate copolymer (1:2, 8% weight/weight) in an ethanol, ethyl acetate, and water (63:30:7) solvent, with and without the addition of two chemical penetration enhancers, dexpanthenol and salicylic acid.^12^ This study revealed that the lacquer formulation with the chemical penetration enhancers added was useful in improving drug delivery to the nail plate after a 15-day, twice-daily application period.^12^ The potency of roflumilast is approximately 25–300 times greater than apremilast, which may allow for greater clinical improvement of psoriatic nails.^13^ However, topical roflumilast is lipophilic, which may limit diffusion across the nail plate.^8,13–16^ This could be mitigated through the development of topical roflumilast formulations that contain hydrophilic and polarized solvents, such as ethanol and acetone, and chemical penetration enhancers.^8,15,16^

Oral apremilast is another option for the treatment of psoriatic nails, with significant and sustained improvement of psoriatic nails observed in clinical studies, and it can also be used to manage other subtypes of psoriasis.^17^ However, systemic side effects of nausea and diarrhea may limit tolerability in some patients.^18^ The use of topical roflumilast for nail psoriasis is a compelling option if patients are able to achieve adequate disease control through this treatment modality, due to the avoidance of systemic side effects. However, further analysis of this treatment is needed to evaluate whether topical roflumilast may be useful in other cases of nail psoriasis, especially in patients with more widespread and severe nail involvement.

This case report suggests that topical roflumilast may be a useful and well-tolerated therapy for the long-term management of both cutaneous and nail manifestations of psoriasis. Further study in randomized controlled trials is indicated to evaluate the local bioavailability and efficacy of topical roflumilast in patients with nail psoriasis. Additionally, the development of roflumilast nail lacquers with chemical penetration enhancers should be explored to optimize the efficacy of topical roflumilast in patients with refractory nail psoriasis.

**Figure 1.**
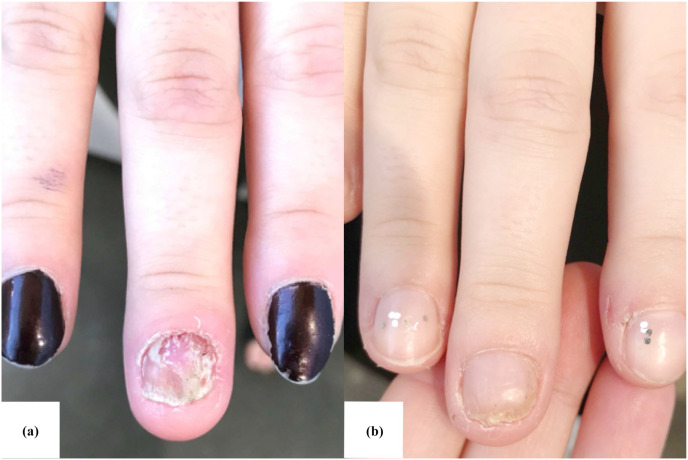
Treatment of nail psoriasis on the right third finger with daily application of topical roflumilast in a 25-year-old female. At baseline (a), the patient presented with onychodystrophy of the right third fingernail, which included findings of onycholysis, a salmon-colored oil drop, and subungual hyperkeratosis. Scaling and erythema of the cuticle, proximal and lateral nail folds, and the distal pulp of the right third finger were also observed. After 5 months of daily application of a topical 0.3% roflumilast cream (b), complete resolution of periungual inflammation and onychodystrophy was achieved.
